# The Effect of Leptin on the Blood Hormonal Profile (Cortisol, Insulin, Thyroid Hormones) of the Ewe in Acute Inflammation in Two Different Photoperiodical Conditions

**DOI:** 10.3390/ijms23158109

**Published:** 2022-07-23

**Authors:** Agata Krawczyńska, Andrzej Przemysław Herman, Hanna Antushevich, Joanna Bochenek, Karolina Wojtulewicz, Dorota Anna Zieba

**Affiliations:** 1 The Kielanowski Institute of Animal Physiology and Nutrition, Polish Academy of Sciences, Instytucka 3, 03-105 Jabłonna, Poland; a.herman@ifzz.pl (A.P.H.); a.antuszewicz@ifzz.pl (H.A.); j.bochenek@ifzz.pl (J.B.); k.wojtulewicz@ifzz.pl (K.W.); 2 Department of Animal Nutrition and Biotechnology, and Fisheries, Faculty of Animal Sciences, University of Agriculture in Krakow, 31-120 Krakow, Poland; rzzieba@cyf-kr.edu.pl

**Keywords:** cortisol, insulin, leptin, lipopolysaccharide, photoperiod, plasma, sheep, stress, thyroid

## Abstract

As a day animal with sensitivity to inflammation similar to that of humans, the sheep may highly outperform the rodent model in inflammation studies. Additionally, seasonality makes sheep an interesting model in endocrinology research. Although there are studies concerning inflammation’s influence on leptin secretion and vice versa, a ewe model, with its possible ‘long-day leptin resistance’, is still not examined enough. The present study aimed to examine whether leptin may modulate an acute inflammation influence on plasma hormones in two photoperiodical conditions. The experiment was conducted on 48 ewes divided into four groups (control, lipopolysaccharide (LPS), leptin, LPS + leptin) during short and long days. Blood sampling started 1 hour before and continued 3 h after LPS/saline administration for further hormonal analysis. The results showed that the photoperiod is one of the main factors influencing the basal concentrations of several hormones with higher values of leptin, insulin and thyroid hormones during long days. Additionally, the acute inflammation effect on cortisol, insulin and thyroid hormones was photoperiod-dependent. The endotoxemia may also exert an influence on leptin concentration regardless of season. The effects of leptin alone on hormone blood concentrations are rather limited; however, leptin can modulate the LPS influence on insulin or thyroxine in a photoperiod-dependent way.

## 1. Introduction

One of the significant factors modulating peripheral leptin concentration is immunological stress induced by bacterial endotoxin administration (lipopolysaccharide, LPS). Previous studies conducted on rodents have shown that LPS increased plasma leptin content and enhanced leptin mRNA expression in adipose tissue, which may be mediated at least in part by interleukin (IL)-1β [1,2]. Wang and Nakayama [3] suggested that the interaction between IL-1β and leptin is mediated by cyclooxygenase-2 (COX-2). IL-1β activates the transcription factor Nuclear Factor κB (NF-κB), which increases the expression of COX-2, which catalyzes the conversion of arachidonic acid to prostaglandin H_2_ (PGH_2_). This, in turn, with the involvement of terminal prostaglandin E synthase, is converted to prostaglandin E_2_ (PGE_2_), which, through one of its receptors belonging to the group of protein G (EP3R), regulates the synthesis of leptin. This is confirmed by the study of Fain et al. [4], who showed that arachidonic acid derivatives modulated the secretion of leptin. The involvement of leptin in LPS-induced fever and anorexia was confirmed by Sachot et al. [5] and Harden et al. [6]. On the contrary, Faggioni et al. [7] stated that leptin per se is not essential for LPS-induced anorexia despite its increase in plasma after LPS injection to leptin receptor-deficient (*db*/*db*) and leptin-deficient (*ob*/*ob*) mice. Additionally, Kim et al. [8] stated that leptin is not involved in acute anorexia caused by LPS injection because, as they observed, the leptin plasma content started to increase after 8 h from LPS treatment. They suggested that insulin, together with neuropeptide Y (NPY), are causative factors of LPS-induced anorexia, as insulin concentrations increased from the second hour after LPS injection.

However, the LPS-induced changes in leptin secretion are confirmed only for rodents. As was observed by Soliman et al. [9] in ewes, no effect of LPS on leptin plasma content was observed even after 12 h from LPS injection. However, during experimental endotoxemia, they observed a rapid increase in cortisol (stress marker) and insulin concentrations and thus suggested insulin as a mediator of observed fever and anorexia. In our opinion, such observation needs to be confirmed, and the possible mechanism of such a relationship should be proposed. It can be supposed that the results obtained by Soliman et al. [9] may be due to the daylength dependency of leptin in ruminants. Unlike most livestock species, sheep are widely known as an animal with a marked seasonality of breeding activity. The annual cycle of the daily photoperiod has been identified as the determinant factor of this phenomenon, while environmental temperature, nutritional status, social interactions, lambing date, lactation period and existing inflammation states are considered to modulate it. The principal biochemical factor connected with the photoperiod is melatonin, which, in ewes, indicates daily and annual changes. As stated by Skipor et al. [10], besides melatonin fluctuations, in ewes, there is an observed seasonal variation in thyroid gland activity to adapt the animal metabolic balance to different environmental conditions, energy requirements and food availabilities. As was shown by Marie et al. [11], the plasma leptin concentration in ewes changed according to the seasons. Moreover, increased leptin concentration during the long-day period was accompanied by elevated daily food intake and body weight and no changes in insulin plasma concentrations. The observed relationship is opposite to the physiological anorexic actions of leptin accompanied by body mass reduction [12]. Adam et al. [13] observed that the intracerebroventricular injection of leptin caused appetite suppression and the stimulation of the frequency of luteinizing hormone (LH) pulses, but only in autumn and not in the spring. This proved that, during a long-day (LD, see explanation in the Materials and Methods) period, sheep become resistant to leptin actions at the hypothalamus level. This is strictly connected with the reproduction cycle in ewes, which are fertile only during a short-day (SD) period. As was pointed out by Chilliard et al. [14] ‘the long-day sheep’ can be treated as a model to study the hyperleptinemic state. Zięba et al. [15] made a further suggestion, proposing ‘long-day ewes’ as a model for obesity research because, similar to obese people, they are characterized by an enhanced food intake and reduced energy expenditure accompanied by high leptin concentrations. The same team also observed that, during a long-day period, resistance is connected with the suppressor of cytokine signalling 3 (SOCS-3) actions at the medio-basal hypothalamus [16,17]. Moreover, our team has reported that the photoperiod affects leptin action on the aorta [18], perivascular adipose tissue (PVAT) [19], choroid plexus [20] and anterior pituitary [21] in ewes challenged with LPS.

Although there are studies concerning inflammation’s influence on leptin secretion, a ewe model with its photoperiod dependence is still not examined enough. Moreover, only a few studies are showing whether leptin can modulate acute inflammation effects, none of which concentrate on a basic hormonal blood profile (cortisol, thyroid hormones and insulin). So, the present study aimed to examine whether leptin may modulate an LPS-induced inflammation influence on plasma hormones in ewes in two different photoperiodical conditions.

## 2. Results

### 2.1. Leptin Plasma Concentration

**Figure 1:**
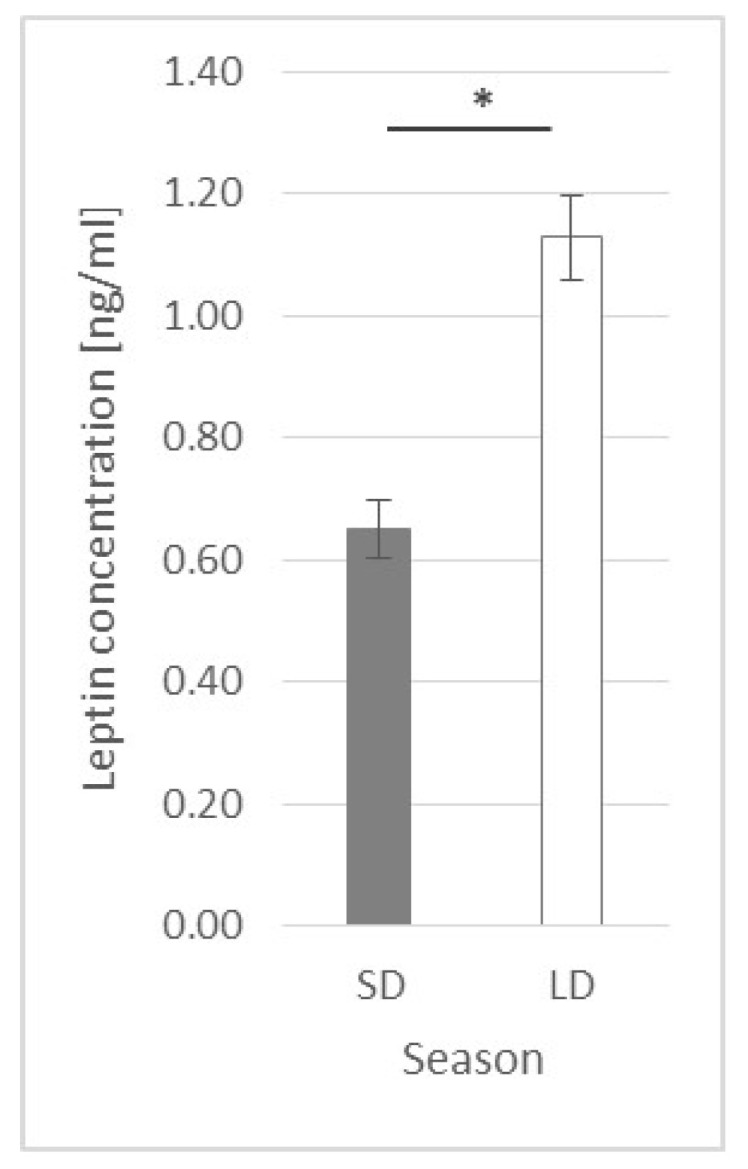
Plasma leptin concentration—comparison between short-day (SD) and long-day (LD) seasons at a 0 h time point; *—indicates *p* ≤ 0.05, according to Student’s *t*-test (*n* = 24).

Generally, the leptin concentration was higher (*p* ≤ 0.0001) in long-day (LD) season animals (1.13 ± 0.07 ng/mL) than that in short-day (SD) season ones (0.65 ± 0.047 ng/mL) (Figure 1).

**Figure 2:**
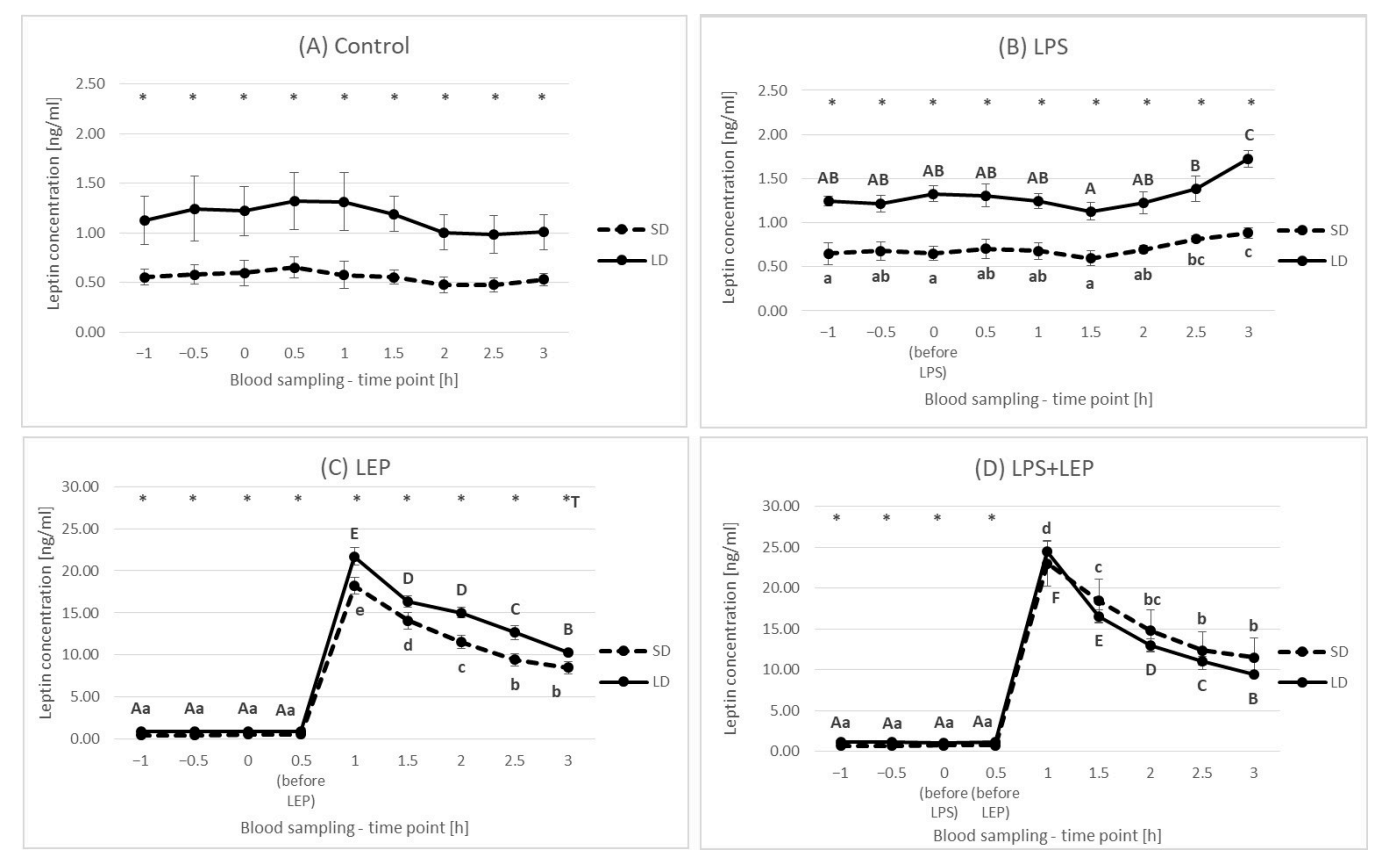
Plasma leptin concentration for each experimental group separately (Control (**A**), LPS (**B**), LEP (**C**) and LPS + LEP (**D**)) at each examined time point and season; *—indicates the statistically significant difference between the short-day (SD) and long-day (LD) photoperiod at each time point according to Student’s *t*-test (*p* ≤ 0.05) (*n* = 6); *T—indicates the tendency in a difference between photoperiods at each time point according to Student’s *t*-test (0.1 > *p* > 0.05); a–e and A–F—indicate the statistically significant difference between time points for the SD and LD seasons, respectively, according to a repeated measured analysis of variance (ANOVA) followed by Fisher’s post-hoc test (*p* ≤ 0.05) (*n* = 6). Groups: control—saline injection, LPS—lipopolysaccharide injection at a dose of 400 ng/kg of body mass after a 0 h time point, LEP—leptin injected at a dose of 20 μg/kg of body mass at a 0.5 h time point, LPS + LEP—LPS and LEP injections at the same doses and time points as those in the LPS and LEP groups.

This was also observed at each time point when the control groups were compared (Figure 2A). In comparison to a 0 h time point, LPS injection increased leptin concentration at 2.5 h and 3 h time points in the SD season or only after 3 h from injection in the LD season (Figure 2B). The leptin injection at a 0.5 h time point caused a rapid increase in plasma leptin concentration regardless of the season, and at 1 h time point, the highest leptin concentrations were observed as 18.20 ± 1.01 and 21.68 ± 1.05 ng/mL for the SD and LD seasons, respectively (Figure 2C). Starting from that moment, the concentration of leptin was systematically falling to the final level of 8.49 ± 0.73 and 10.28 ± 0.30 ng/mL for the SD and LD seasons, respectively; however, at each time point, the leptin concentration was higher in the LD season than that in the SD season. When leptin was injected after LPS, a rapid increase in leptin concentration was also observed, followed by a systematic decrease (Figure 2D).

**Figure 3:**
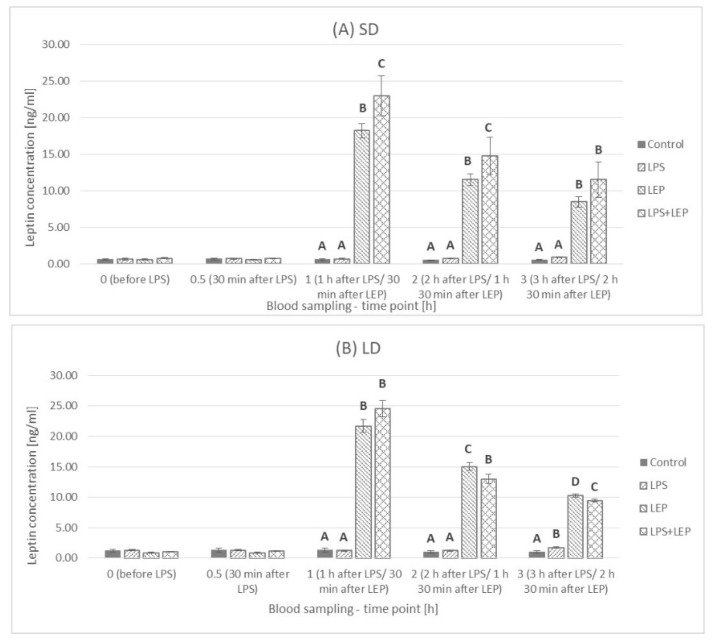
Plasma leptin concentration—comparison among the experimental groups (Control, LPS, LEP and LPS + LEP) for short-day (SD) (**A**) and long-day (LD) (**B**) photoperiod for each selected time point separately; A–D—bars with different letters differ significantly within each time point according to the two-way analysis of variance (ANOVA) followed by Fisher’s post-hoc test (*p* ≤ 0.05) (*n* = 6). Groups: control—saline injection, LPS—lipopolysaccharide injection at a dose of 400 ng/kg of body mass at a 0 h time point, LEP—leptin injected at a dose of 20 μg/kg of body mass at a 0.5 h time point, LPS + LEP—LPS and LEP injections at the same doses and time points as those in the LPS and LEP groups.

When the experimental groups from the SD season were compared (Figure 3A), it was observed that, starting from a 1 h time point, a significant effect of leptin was observed (*p* ≤ 0.0001 for 1 h, 2 h and 3 h time points). Although the leptin concentration was higher in the LPS + LEP group than in the LEP group, only the tendency in the interaction between leptin and LPS was observed at a 1 h time point (*p* = 0.063). Additionally, when the experimental groups from the LD season were compared (Figure 3B), it was stated that, starting from a 1 h time point, a significant effect of leptin was observed (*p* ≤ 0.0001 for 1 h, 2 h and 3 h time points). However, in the LD season, the leptin concentration was lower in the LPS + LEP group than that in the LEP group at both 2 h and 3 h time points. Moreover, the significant interaction between LPS and leptin was observed at a 3 h time point (*p* = 0.004), showing that, although LPS alone increased leptin concentration (increase in the LPS group vs. the control one), after leptin injection, its effect was not observed (a higher leptin concentration in the LEP group than that in the LPS + LEP group).

### 2.2. Cortisol Plasma Concentration

**Figure 4:**
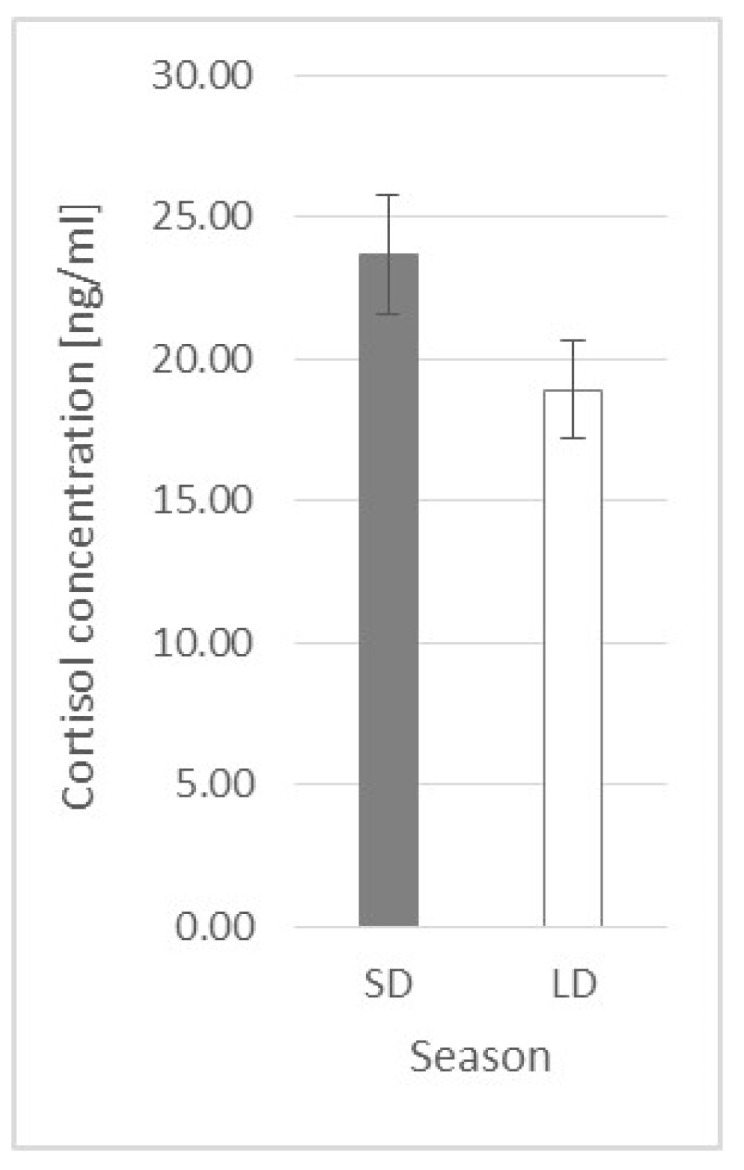
Plasma cortisol concentration—comparison between short-day (SD) and long-day (LD) seasons at a 0 h time point (*n* = 24).

Generally, there was no difference in cortisol concentration between seasons (*p* = 0.14), and it was 23.66 ± 2.105 ng/mL and 18.91 ± 1.758 ng/mL for SD and LD, respectively (Figure 4).

**Figure 5:**
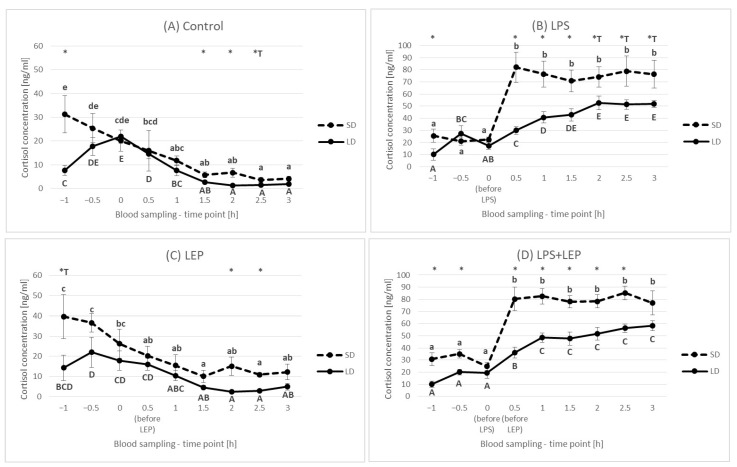
Plasma cortisol concentration for each experimental group separately (Control (**A**), LPS (**B**), LEP (**C**) and LPS + LEP (**D**)) at each examined time point and season; *—indicates the statistically significant difference between the short-day (SD) and long-day (LD) photoperiods at each time point according to Student’s *t*-test (*p* ≤ 0.05) (*n* = 6); *T—indicates the tendency in a difference between photoperiods at each time point according to Student’s *t*-test (0.1 > *p* > 0.05); a–e and A–E—indicate the statistically significant difference between time points for the SD and LD seasons, respectively, according to a repeated measured analysis of variance (ANOVA) followed by Fisher’s post-hoc test (*p* ≤ 0.05) (*n* = 6). Groups: control—saline injection, LPS—lipopolysaccharide injection at a dose of 400 ng/kg of body mass after a 0 h time point, LEP—leptin injected at a dose of 20 μg/kg of body mass at a 0.5 h time point, LPS + LEP—LPS and LEP injections at the same doses and time points as those in the LPS or LEP groups.

At a 0 h time point, there were no differences between the seasons that were observed during the first adaptation hour (Figure 5). In the control groups, the cortisol concentration decreased, and starting from the 1.5 h time point, it was stable regardless of the season (Figure 5A); however, at 1.5 h, 2 h and 2.5 h time points, the cortisol concentration was higher in animals from the SD season. The LPS injection in the SD season caused a rapid increase in the cortisol level, which did not change from the 0.5 h time point to the 3 h time point (Figure 5B); however, in the LD season, the cortisol concentration increased more slowly, stabilizing after 1.5 h, but still, the values obtained in the LD season were lower than the values obtained in the SD season. Leptin injection did not modify the pattern of cortisol concentration changes in comparison to the control groups (Figure 5C). Leptin injection also did not influence the LPS effect on the cortisol profile (Figure 5D).

**Figure 6:**
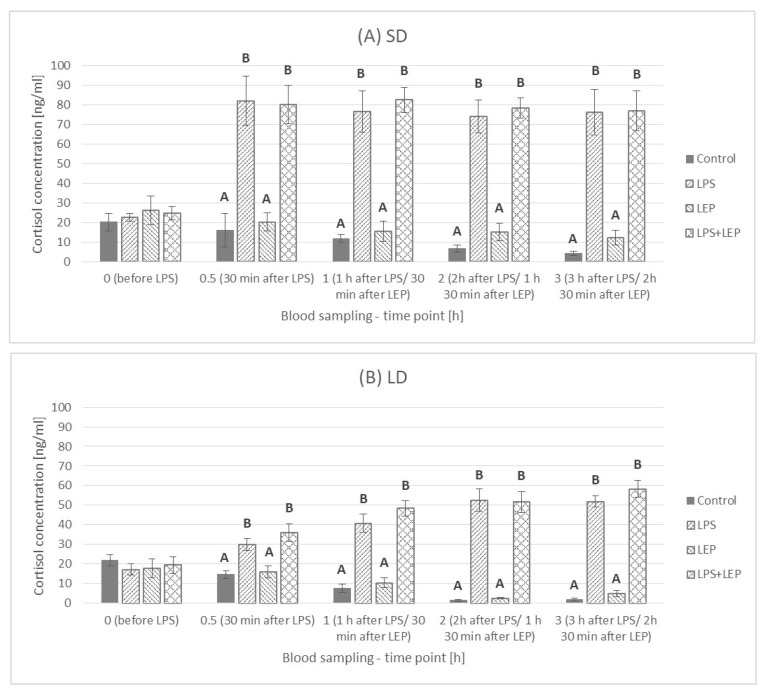
Plasma cortisol concentration—comparison among the experimental groups (control, LPS, LEP and LPS + LEP) for short-day (SD) (**A**) and long-day (LD) (**B**) photoperiods for each selected time point separately; A,B—bars with different letters differ significantly within each time point according to the two-way analysis of variance (ANOVA) followed by Fisher’s post-hoc test (*p* ≤ 0.05) (*n* = 6). Groups: control—saline injection, LPS—lipopolysaccharide injection at a dose of 400 ng/kg of body mass at a 0 h time point, LEP—leptin injected at a dose of 20 μg/kg of body mass at a 0.5 h time point, LPS + LEP—LPS and LEP injections at the same doses and time points as those in the LPS and LEP groups.

When the experimental groups from either the SD or LD season were compared (Figure 6A,B), only the LPS effect was observed (*p* ≤ 0.0001 for 0.5 h, 1 h, 2 h and 3 h time points for both seasons). No interaction between LPS and leptin was observed.

### 2.3. Insulin Plasma Concentration

**Figure 7:**
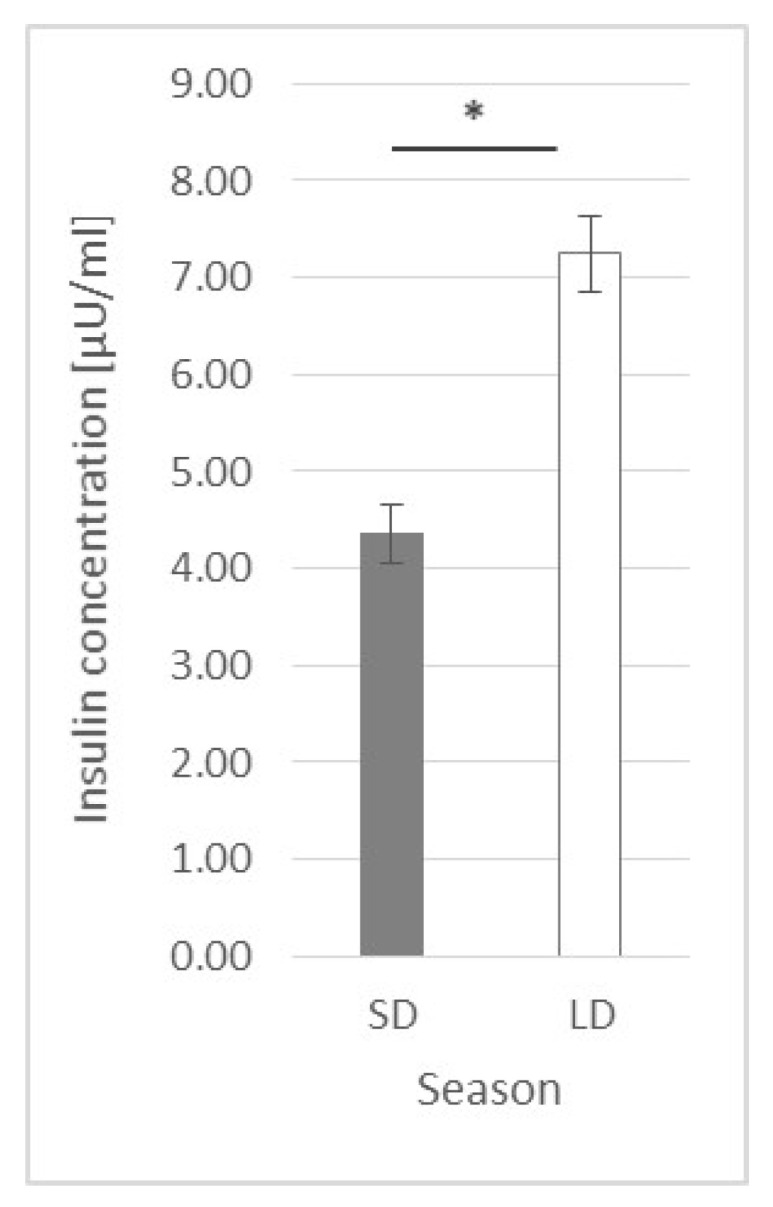
Plasma insulin concentration—comparison between short-day (SD) and long-day (LD) seasons at a 0 h time point; *—indicates *p* ≤ 0.05, according to Student’s *t*-test (*n* = 24).

Generally, the insulin concentration was higher (*p* ≤ 0.0001) in the LD season animals (7.25 ± 0.386 μU/mL) than that in the SD season ones (4.36 ± 0302 μU/mL) (Figure 7).

**Figure 8:**
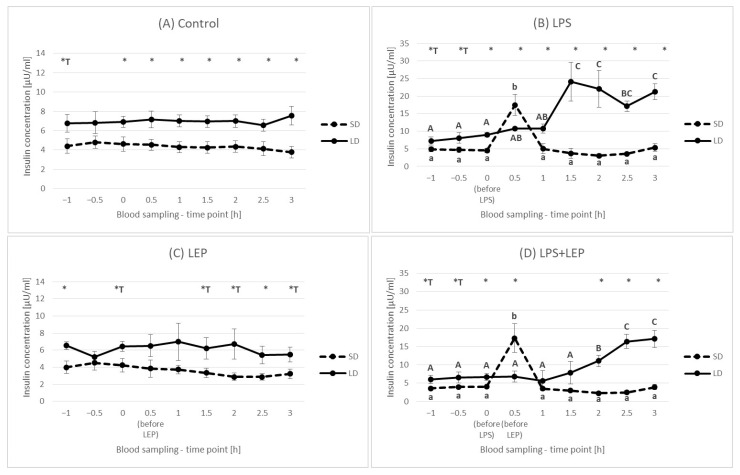
Plasma insulin concentration for each experimental group separately (Control (**A**), LPS (**B**), LEP (**C**) and LPS + LEP (**D**)) at each examined time point and season; *—indicates the statistically significant difference between the short-day (SD) and long-day (LD) photoperiods at each time point according to Student’s *t*-test (*p* ≤ 0.05) (*n* = 6); *T—indicates the tendency in a difference between photoperiods at each time point according to Student’s *t*-test (0.1 > *p* > 0.05); a–b and A–C—indicate the statistically significant difference between time points for the SD and LD seasons, respectively, according to a repeated measured analysis of variance (ANOVA) followed by Fisher’s post-hoc test (*p* ≤ 0.05) (*n* = 6). Groups: control—saline injection, LPS—lipopolysaccharide injection at a dose of 400 ng/kg of body mass after a 0 h time point, LEP—leptin injected at a dose of 20 μg/kg of body mass at a 0.5 h time point, LPS + LEP—LPS and LEP injections at the same doses and time points as those in the LPS or LEP groups.

This was also observed at each time point, except for the –0.5 h time point, when the control groups were compared (Figure 8A). In comparison to the 0 h time point, LPS injection rapidly increased insulin concentration only at the 0.5 h time point in the SD season; however, in the LD season, LPS increased the insulin concentration from the 1.5 h time point, and it was kept at the same level or higher compared to the SD season level until the end of the experiment (Figure 8B). The leptin injection did not influence the insulin concentration in time (Figure 8C). When leptin was injected after LPS in the SD season, it did not inhibit the LPS effect on insulin, which was still a one-time-point pick (Figure 8D). During the LD season, an increase in the insulin concentration in the LPS + LEP group was observed from the 2 h time point, and it was the highest at the 2.5 h and 3 h time points.

**Figure 9:**
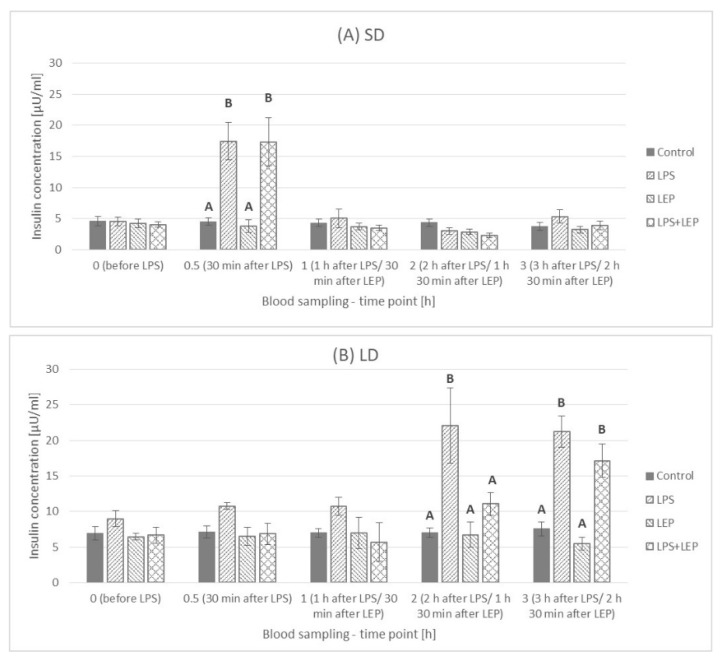
Plasma insulin concentration—comparison among the experimental groups (Control, LPS, LEP and LPS + LEP) for the short-day (SD) (**A**) and long-day (LD) (**B**) photoperiods for each selected time point separately; A,B—bars with different letters differ significantly within each time point according to the two-way analysis of variance (ANOVA) followed by Fisher’s post-hoc test (*p* ≤ 0.05) (*n* = 6). Groups: control—saline injection, LPS—lipopolysaccharide injection at a dose of 400 ng/kg of body mass at a 0 h time point, LEP—leptin injected at a dose of 20 μg/kg of body mass at a 0.5 h time point, LPS + LEP—LPS and LEP injections at the same doses and time points as those in the LPS or LEP group.

When the experimental groups from the SD season were compared at selected time points (Figure 9A), the LPS effect was observed at a 0.5 h time point (*p* ≤ 0.0001), and the insulin increase was to the same level in both the LPS and LPS + LEP groups. On the other hand, in the LD season (Figure 9B), the LPS effect was observed at 2 h and 3 h time points (*p* ≤ 0.003 and *p* ≤ 0.0001, respectively). However, the leptin injection after LPS delayed the insulin concentration increase, as the LPS + LEP group did not differ from the control or LEP groups at a 2 h time point, but such a difference was noticed after 3 h (tendency in the interaction between LPS and leptin at a 2 h time point; *p* = 0.062).

### 2.4. Thyroxine (T4) Plasma Concentration

**Figure 10:**
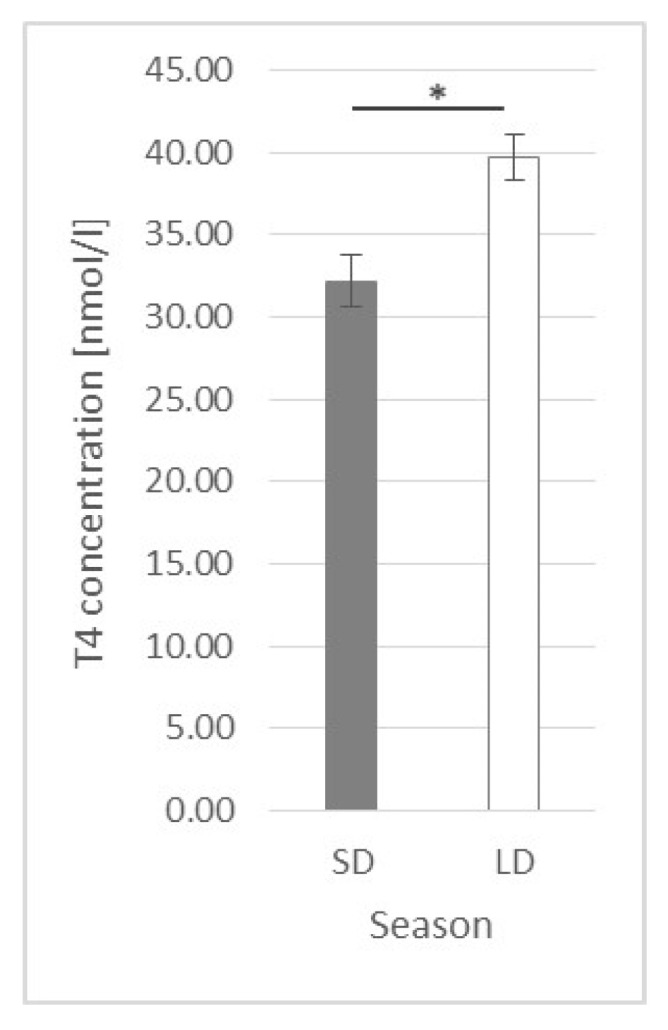
Plasma thyroxine (T4) concentration—comparison between short-day (SD) and long-day (LD) seasons at a 0 h time point; *—indicates *p* ≤ 0.05, according to Student’s *t*-test (*n* = 24).

Generally, the T4 concentration was higher (*p* ≤ 0.002) in the LD season animals (39.75 ± 1.413 nmol/L) than that in the SD season ones (32.21 ± 1.591 nmol/L) (Figure 10).

**Figure 11:**
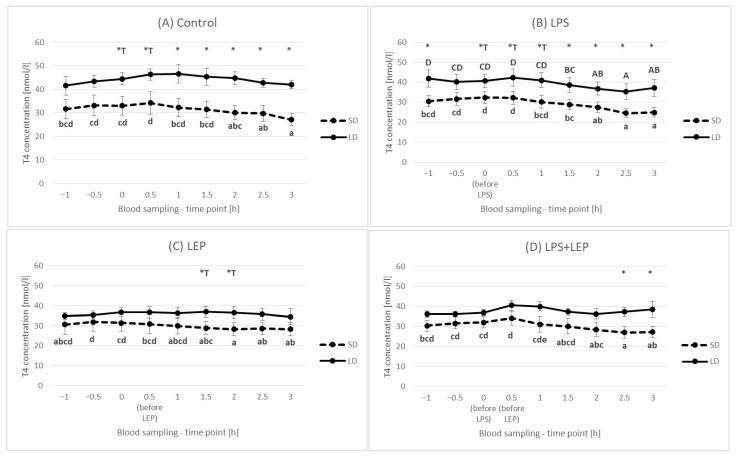
Plasma thyroxine (T4) concentration for each experimental group separately (Control (**A**), LPS (**B**), LEP (**C**) and LPS + LEP (**D**)) at each examined time point and season; *—indicates the statistically significant difference between the short-day (SD) and long-day (LD) photoperiods at each time point according to Student’s *t*-test (*p* ≤ 0.05) (*n* = 6); *T—indicates the tendency in a difference between photoperiods at each time point according to Student’s *t*-test (0.1 > *p* > 0.05); a–d and A–D—indicate the statistically significant difference between time points for the SD and LD seasons, respectively, according to a repeated measured analysis of variance (ANOVA) followed by Fisher’s post-hoc test (*p* ≤ 0.05) (*n* = 6). Groups: control—saline injection, LPS—lipopolysaccharide injection at a dose of 400 ng/kg of body mass after a 0 h time point, LEP—leptin injected at a dose of 20 μg/kg of body mass at a 0.5 h time point, LPS + LEP—LPS and LEP injections at the same doses and time points as those in the LPS and LEP groups.

It was observed that, in the control group, the T4 concentration decreased in time, but only in the SD season (lower values at the 2.5 h and 3 h time points in comparison to the 0 h time point) (Figure 11A). When LPS was injected, the observed SD season control group time-dependent decrease became more pronounced in the SD season, but it was also observed in the LD season (Figure 11B). In the leptin-treated SD group, a time-dependent decrease was observed; however, this was not the case in the LD season (Figure 11C). A similar relationship was observed for the LPS + LEP groups (Figure 11D), which suggests that leptin injection inhibited the LPS effect.

**Figure 12:**
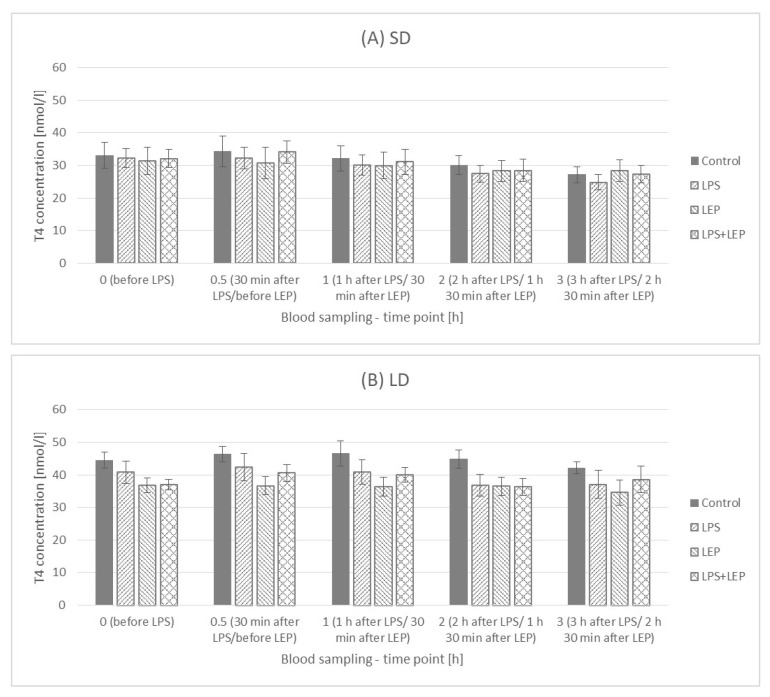
Plasma thyroxine (T4) concentration—comparison among the experimental groups (control, LPS, LEP and LPS + LEP) for the short-day (SD) (**A**) and long-day (LD) (**B**) photoperiods for each selected time point separately; *n* = 6. Groups: control—saline injection, LPS—lipopolysaccharide injection at a dose of 400 ng/kg of body mass at a 0 h time point, LEP—leptin injected at a dose of 20 μg/kg of body mass at a 0.5 h time point, LPS + LEP—LPS and LEP injections at the same doses and time points as those in the LPS or LEP groups.

When the experimental groups from either the SD or LD season were compared (Figure 12A,B), neither an LPS nor leptin effect was observed. Additionally, no interaction between LPS and leptin was observed.

### 2.5. Triiodothyronine (T3) Plasma Concentration

**Figure 13:**
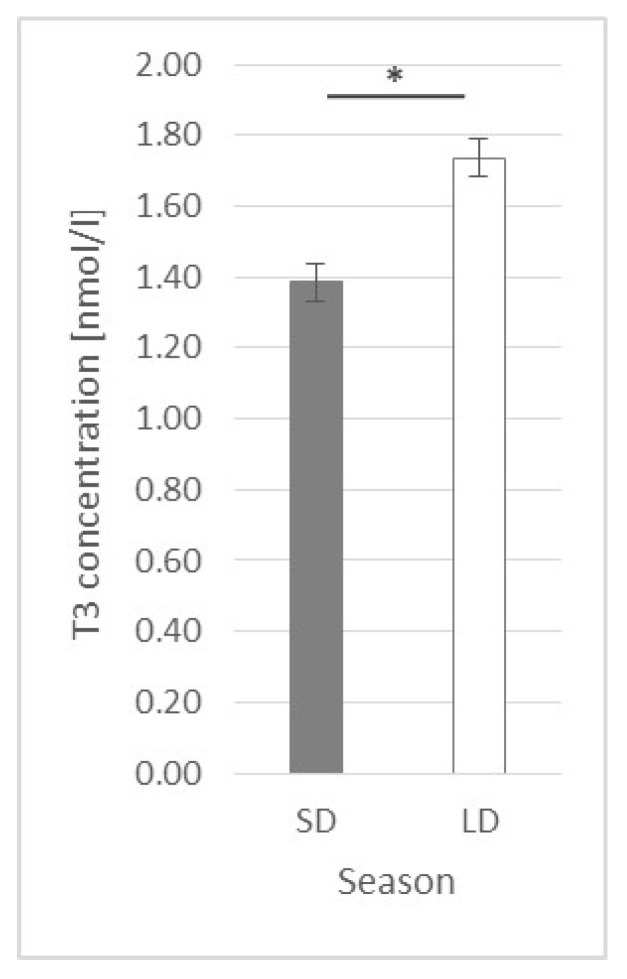
Plasma triiodothyronine (T3) concentration—comparison between the short-day (SD) and long-day (LD) seasons at a 0 h time point; *—indicates *p* ≤ 0.05, according to Student’s *t*-test (*n* = 24).

Generally, the T3 concentration was higher (*p* ≤ 0.0002) in the LD season animals (1.74 ± 0.055 nmol/L) than that in the SD season ones (1.38 ± 0.052 nmol/L) (Figure 13).

**Figure 14:**
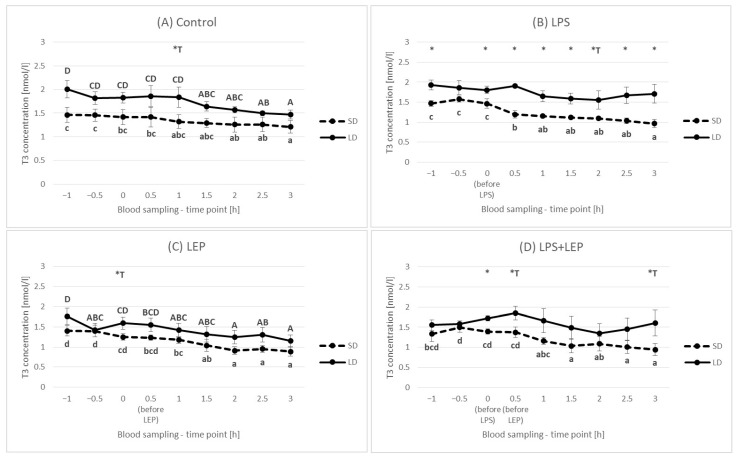
Plasma triiodothyronine (T3) concentration for each experimental group separately (control (**A**), LPS (**B**), LEP (**C**) and LPS + LEP (**D**)) at each examined time point and season; *—indicates the statistically significant difference between the short-day (SD) and long-day (LD) photoperiods at each time point according to Student’s *t*-test (*p* ≤ 0.05) (*n* = 6); *T—indicates the tendency in a difference between photoperiods at each time point according to Student’s *t*-test (0.1 > *p* > 0.05); a–d and A–D—indicate the statistically significant difference between the time points for the SD and LD seasons, respectively, according to a repeated measured analysis of variance (ANOVA) followed by Fisher’s post-hoc test (*p* ≤ 0.05) (*n* = 6). Groups: control—saline injection, LPS—lipopolysaccharide injection at a dose of 400 ng/kg of body mass after a 0 h time point, LEP—leptin injected at a dose of 20 μg/kg of body mass at a 0.5 h time point, LPS + LEP—LPS and LEP injections at the same doses and time points as those in the LPS or LEP group.

It was observed that, in the control group, the T3 concentration decreased in time (at a 3 h time point in the SD season and at 2.5 h and 3 h time points in the LD season) in comparison to the 0 h time point (Figure 14A). When LPS was injected, the observed control groups decrease became more pronounced in the SD season (decrease from a 0.5 h time point), but it was inhibited in the LD season (Figure 14B). Regardless of the season, leptin injection did not diminish the T3 decrease and even pronounced it (Figure 14C). However, when leptin was injected after prior LPS stimulation, the T3 concentration changes were blocked in the LD season but not in the SD season (Figure 14D).

**Figure 15:**
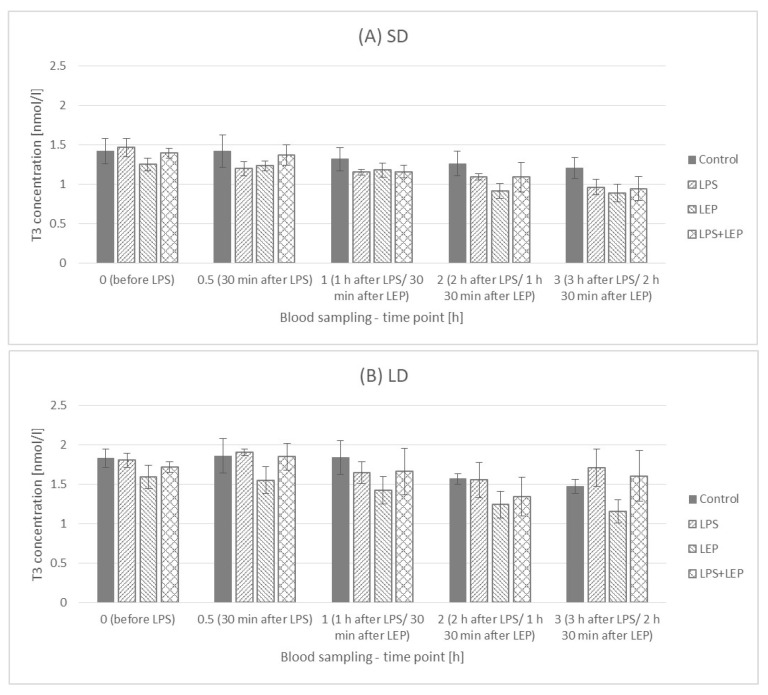
Plasma triiodothyronine (T3) concentration—comparison among the experimental groups (control, LPS, LEP and LPS + LEP) for the short-day (SD) (**A**) and long-day (LD) (**B**) photoperiods for each selected time point separately; *n* = 6. Groups: control—saline injection, LPS—lipopolysaccharide injection at a dose of 400 ng/kg of body mass at a 0 h time point, LEP—leptin injected at a dose of 20 μg/kg of body mass at a 0.5 h time point, LPS + LEP—LPS and LEP injections at the same doses and time points as those in the LPS and LEP groups.

When the experimental groups from either the SD or LD season were compared (Figure 15A,B), neither an LPS nor leptin effect was observed. Additionally, no interaction between LPS and leptin was observed.

## 3. Discussion

The problem of hormonal and metabolic disorders with stress dependence is of more and more concern these days. Leptin resistance, insulin resistance, hypothyroidism or increased concentrations of cortisol in the blood are only the basic disorders that people, especially young women of reproductive age, have to manage [22,23,24].

### 3.1. Leptin Plasma Concentration

Stress is a state of imbalanced homeostasis during which a variety of adaptive processes are activated that cause physiological and behavioural changes. The animal research on stress influence is mostly based on rodents; however, such an animal model is not a perfect one, as rats/mice are nocturnal animals with a much faster metabolism (for example, LPS metabolism) than humans. So, new animal models are searched for. Ewe, which is already a widely used model in neuroendocrinological studies, seems to also be a good model in metabolic disorders and immunological studies. Ewes not only have a human-like sensitivity to LPS (gene level [25]; absolute lethal dose for sheep—0.025 mg LPS/kg; human shock dose—0.015 mg LPS/kg; mice median lethal dose—10 mg LPS/kg [26]) but are also characterized by a natural leptin resistance resulting from the change in the length of the day [27]. However, the influence of inflammation on leptin secretion in the content of photoperiod dependence is not well examined. In ewes, only Soliman et al. [9] stated that there was no effect of intravenous (*i.v.*) LPS injection on leptin plasma concentration, even after 12 h from LPS injection. However, the authors did not indicate in which photoperiod the study was conducted. Additionally, in cows, Soliman et al. [28] did not observe the LPS effect on leptin concentration. In the present study, it was observed that, regardless of the photoperiod, the leptin blood concentration started to increase 3 h after LPS *i.v.* injection. Discrepancies between the study by Soliman et al. [9] and the present study are difficult to explain, especially when the used doses are rather similar (450 vs. 400 ng/kg of body mass). However, Soliman conducted the experiment on younger sheep (only 6–9 months old). As no other studies are showing the LPS effect on leptin in ewes, the discrepancies should be verified in the future; moreover, differences between acute and prolonged stress should be studied. On the other hand, the increase in leptin concentration that was observed in the present study after LPS is consistent with studies on rodents and humans [1,2,8]. To our knowledge, it is also the first study showing the lack of a photoperiod effect on LPS changes in the leptin concertation. However, it must be remembered that leptin concentration in ewes is strictly connected with the photoperiod. In the present study, the leptin concentration was higher in the LD season animals (1.13 ± 0.07 ng/mL) than that in the SD season ones (0.65 ± 0.047 ng/mL). Even higher differences between seasons were observed by Marie et al. [11], who stated that, during the LD period, the leptin concentration was elevated by more than three times (7 vs. 2 ng/mL for the SD and LD seasons, respectively).

In the present study, we examined if additional leptin infusion would influence its own metabolism in an acute inflammation state. So, the possible differences were inspected when leptin was injected alone and after LPS. The lone leptin injection increased its plasma level, but still, the higher content observed during the LD season was stated here. When a prior LPS injection was made, there was no difference in the leptin peak concentration, but the leptin concentration decreased faster when a prior LPS injection was carried out in comparison to animals in the physiological state, which may suggest that either leptin had a faster metabolism or it bounded faster to its receptor to exert the effect. Moreover, leptin can bind not only to its receptor but also non-specifically to the IL-6 receptor (IL-6R) [29], and it is well documented that IL6-R expression is highly increased after LPS injection as well, which we also observed in acute stress conditions in sheep aorta [18], perivascular adipose tissue [19], hypothalamus [30] and anterior pituitary [21], but not in the prolonged inflammation in the anterior pituitary [31].

### 3.2. Cortisol Plasma Concentration

Cortisol is one of the main indicators of an organism response to stressful conditions. The increase in cortisol level is also well documented in inventory animals such as ewes [32,33]; however, neither a photoperiod nor leptin influence is described in the literature. In the present study, no effect of leptin on the cortisol concentration in blood was observed. At the basing time point, no statistically significant seasonal differences were observed (23.66 ± 2.105 and 18.91 ± 1.758 ng/mL for SD and LD, respectively). However, in humans, a seasonal variation in human salivary cortisol concentration was observed, with the highest concentrations observed in February, March and April and the lowest ones observed in July and August [34]. The seasonal changes in cortisol were also observed in Bedouin goats [35], with the highest levels occurring in the summer and winter when the environmental conditions are at their extreme levels; however, it must be stressed that the research was conducted in an extremely hot climate typical for deserts. In the present study, there was no difference in cortisol concentration between the groups at a 0 h time point regardless of the season, which suggests that, after a one-hour adaptation period, the animals calmed down and got used to the people handling them. Regardless of the season, in the control groups and leptin-treated groups, the cortisol level slowly decreased to the end of the experiment, while in the LPS-treated groups with or without leptin treatment, the cortisol level increased. However, the LPS influence was highly dependent on the season. During the SD period, the observed increase was rapid (observed 30 min after LPS injection) and remained at the same high level to the end of the experiment. In the LD season, the cortisol increase was not so steep but was spread over time. The cortisol concentration during the LD season also did not reach values as high as those reached during the SD season (except for the LPS + LEP groups at a 3 h time point, which did not differ). Such seasonal differences may suggest that animals in different seasons answer to stress differently as the hypothalamic-pituitary-adrenal axis exerts important anti-inflammatory and immune-modulating actions [36]. The acute increase in adrenal glucocorticoid secretion in response to infection is therefore thought to be an adaptive homeostatic mechanism to prevent immunological over-reaction [37] caused by acute rapid proinflammatory cytokine secretion. Following this line of reasoning, lower blood cortisol concentrations in the SD season could mean that the release of pro-inflammatory cytokines during this season will be greater, as the inhibition of their production will be lower. Some of these observations are in line with our previously published results. We have shown that, in PVAT, *i.v.* LPS administration resulted in a 280-fold increase in the expression of the *IL6* gene in SD and only a 140-fold increase in LD [19]. However, in the pituitary, these differences were even more pronounced because LPS injection caused a 600-fold and 200-fold increase in *IL6* gene expression and a 10-fold and 2-fold increase in *IL1* gene expression for SD and LD, respectively [21]. However, we did not observe such differences at the level of gene expression in the aorta [18]. So, it can be concluded that, in the SD season, the inflammation reaction effects can be more pronounced as hypothalamic-pituitary-adrenal axis activity is inhibited. Whereas the obtained results showed that photoperiod plays an important role in the progression of LPS effects, leptin did not modulate the LPS effect on cortisol changes, which may suggest that leptin participation in inflammation progression is not through the cortisol connected pathway.

### 3.3. Insulin Plasma Concentration

One of the hormones very often mentioned in the context of leptin as well as inflammation is insulin. In the present study, it was observed that the influence of LPS on insulin blood concentration is photoperiod-dependent. In the SD season, an abrupt but short-lasting change was observed, while during the LD season, the inflammation effect was delayed but lasted until the end of the experiment. In the previous experiment, Soliman et al. [9] stated that, during acute endotoxemia in sheep, the insulin blood concentration was increased 2, 4 and 6 h after LPS injection, with a significant decrease to the basal level after 12 h. Such results are similar to the results obtained in the present research during the LD season. Perhaps the elongation of the current study (which was now completed after 3 h) would also show a drop in the concentration of insulin in the blood after a few extra hours. Soliman et al. [9] presented that an increased insulin blood concentration was accompanied by a significant decrease in glucose concentration starting 4 h after LPS injection. A decrease in glucose concentration was also observed by Cadaret et al. [38] in wether lambs following an acute immune challenge; however, the observed fall was preceded by a brief spike in glucose concentration 2 h after LPS injection. Unfortunately, in the present study, no glucose analyses were conducted. The importance of insulin in the LPS-anorectic effect was also stressed by Kim et al. [8], who stated that, in rats, the insulin levels increased from the second hour after LPS injection, but the glucose concentrations were elevated at 8 and 16 h in LPS-treated animals. Moreover, they observed that LPS-induced anorexia was attenuated in insulin-deficient STZ rats and was abolished by insulin treatment; so, they concluded that, in LPS-induced anorexia, insulin may constitute a newly found causative factor, whereas leptin appears to play a minor role in an early period in rats. The photoperiod-dependent differences in insulin concentration after LPS treatment in the present study, as well as the simultaneously higher insulin blood concentration in the LD season, can provide some explanation for the divergent results in the clinical use of insulin in acute stress cases. The problem of acute insulin resistance following injuries and infections has been widely studied [39]. It is well known that acute insulin resistance in humans is followed by hyperglycemia, as both the inability of insulin to adequately stimulate glucose uptake, mainly into skeletal muscle, and the inhibition of gluconeogenesis in the liver occur. To prevent hyperglycaemia, patients in critical states are administered insulin. However, there are groups of patients who do not benefit or are even in danger from intensive insulin therapy [40]. The results of the present study may translate such cases and stress the importance of a simultaneous balance of other hormones (such as leptin) in the organism before such insulin treatment is implemented. So, the photoperiod-dependent LPS effects observed in sheep may be of high interest and point to sheep as an interesting model in the research on the use of insulin in the states related to the occurrence of acute stress reactions, considering the natural leptin resistance state in this animal during the LD season. The potency of insulin usage in inflammation in sheep was studied by Chalmeh et al. [41], who observed that the *i.v.* injection of insulin may have a potential anti-inflammatory effect in Iranian fat-tailed ewes with LPS-induced endotoxemia. In comparison to Soliman et al. [9], the authors stated that the glucose concentration in LPS-treated ewes was increased 4, 5 and 6 h after inflammation induction, which may indicate acute insulin resistance. However, the authors conducted the experiment only in one season, April, which is a rather non-breeding season in the Badjgah region of Iran, with a semiarid climate [42], and should thus correspond to the ‘long-day sheep model’. However, Chalmeh et al. [41] presented no results for the SD season (breeding season), in which the results could be significantly different. Assuming that the results obtained by Chalmeh et al. [41] present the results obtained for LD sheep (a natural model for leptin resistance in obesity), it could be concluded that, in acute conditions, insulin should be administered to people with leptin resistance problems or obese people but not to healthy people. This would also be in line with the results obtained in the present study, in which there was only a long and chronic spike in blood insulin concentration in the LD period. The above statement is quite strong and requires further research to confirm its validity, especially knowing that, in obese people, chronic insulin resistance can occur. In addition, based on the results of the present study, it should be noted that, during the LD period in sheep with acute endotoxemia, the additional *i.v.* administration of leptin caused the increase in insulin concentration to be more gradual and spread over time. Such a leptin effect observed during the LD period may bring into question the occurrence of leptin resistance in this photoperiod. However, in the absence of a blood glucose concentration analysis, it is also difficult to see how the effect of leptin on insulin concentration affected glucose and the potential hyperglycemic state in an acute state of infection.

In the current experiment, the singular leptin *i.v.* injection did not influence blood insulin concentration; however, previously, we found that, in cattle, the effect of peripherally administered ovine leptin (doses of 0.2, 2.0, 20 µg/kg body mass) on insulin secretion was dose-dependent [43]. Although doses of 0.2 and 20 µg/kg of leptin increased circulating insulin briefly, the intermediate dose of leptin (2.0 µg/kg) elevated plasma insulin concentrations for at least 3 h. Some of the studies conducted on rodents or human cell lines showed the inhibitory effect of leptin on proinsulin gene expression [44,45,46]. Pancreatic cells express leptin receptors, and the triggered signaling pathway is the Janus kinase 2 (JAK2) signal transducer and the activator of the transcription 3 (STAT3) pathway [47]. Moreover, it was proposed that leptin acts on pancreatic cells via the hyperpolarization of cells that are activated through ATP-sensitive potassium channels [48]. Leptin can also affect pancreatic endocrine functions through the sympathetic nervous system [49]. However, one of the possible mechanisms by which leptin might have modulated LPS effects on blood insulin concentration during the LD season in the present study can be the exertion of an effect on the LPS main receptor—Toll-like receptor 4 (TLR4)—expression via the JAK2-STAT3 pathway. It was presented that TLR4 is expressed in pancreatic β-cells [50]. Although we did not find any article about the effect of leptin on TLR4 expression in the pancreas, Jiang et al. [51] observed that leptin induces TLR4 expression via the JAK2-STAT3 pathway in obesity-related osteoarthritis, so the leptin effect on TLR4 expression in other organs cannot be excluded. Moreover, Jiang et al. [51] also presented that the silencing of the suppressor of cytokine signaling 3 (SOC3, the main inhibitor of leptin action) significantly influenced TLR4 expression, which can emphasize the leptin-TLR4 dependence. Certainly, the obtained results show that the leptin-insulin-acute inflammation relationship is complicated and requires further intensive research.

### 3.4. Thyroid Hormones Plasma Concentrations

When inflammation issues are discussed in relation to metabolic hormones, the issue of thyroid hormones should not be omitted. As was observed by Skipor et al. [10], and also in the present study, the T3 and T4 hormone levels are higher during the LD season. It can be said that this is a natural adaptation of the animal metabolism to different environmental conditions, energy requirements and food availabilities. It is well known that photoperiod-dependent melatonin fluctuations influence pars tuberalis activity, exerting an effect on hypothalamus thyroid-stimulating hormone (TSH) secretion [52]. As reviewed by Ebling [53], in most species (including sheep), during the SD photoperiod, the gene expression of the two most important enzymes participating in thyroid hormone metabolism, deiodinase 2 (DIO2) and deiodinase (DIO3), is changed (DIO2 is decreased and DIO3 is increased) in the third ventricle in comparison to the LD photoperiod, which results in a catabolic state. DIO2 converts inactive T4 to active T3, while DIO3 inactivates T4 by converting it into reverse T3 (rT3) and degrades T3 into T2. Additionally, the inflammation state is said to influence DIO2 gene expression in tanycytes [54]. However, the matter of the LPS effect in a photoperiod-dependent manner is not widely examined, especially in ewes with possible LD leptin resistance.

In the present study, it was observed that T4 concentration systematically decreased during the experimental hours in the SD season control group and that neither leptin nor LPS treatment influenced this effect. Such observation may suggest that either T4 conversion into T3 has taken place, accompanied by increased DIO2 activity, or T4 was inactivated into rT3 by increased DIO3 activity. On the other hand, in the LD season control group, the T4 concentration was stable, but the LPS caused a time decrease in the T4 concentration (thus inducing the same analogical changes as those observed in the SD season); however, leptin administration blocked the LPS effect. Such data may suggest that the activity of both DIO2 and DIO3 is photoperiod-dependent in ewes, but LPS can significantly modify the photoperiod effect. The effect of leptin observed in the LD season shows its anti-LPS actions; however, why such an effect was observed only in LD is difficult to explain. Perhaps, as with insulin, the mechanism is related to the leptin-TLR4 dependence at the hypothalamus and/or thyroid gland levels; however, further studies are required.

Knowing that both the photoperiod and LPS administration influence T4 concentration, we examined the T3 concentration changes. The changes profile was a little bit different here. The T3 concentration systematically decreased during the experimental hours in both control groups regardless of the season. So, this shows that, in the SD season, a decrease in T4 was accompanied by a decrease in T3, while in the LD season, changes were stated only in T3, but the effect of LPS on T3 concentration was also photoperiod-dependent, as was observed for T4 concentration. However, in the case of T3 concentration, the LPS treatment inhibited its gradual decrease, keeping the T3 level at the basal level. So, it can be stated that only in the LD season does LPS administration cause a decrease in T4 concentration while stabilizing T3 concentration. Such observation may suggest that LPS increased DIO2 activity, which accelerated T4 to T3 conversion, preventing further T3 degradation, which is consistent with the findings of Fekete et al. [54]. Surprisingly, no effect of leptin with or without prior LPS treatment on T3 concentration was observed, regardless of the season, whereas in the LD season, we might have expected leptin to retain the LPS effect, as was the case with T4. However, it should also be added that, although no effect of leptin alone was observed in the current study, our previous in vitro study presented an interaction between leptin, season and thyroid hormones [55]. The results of that study showed that leptin stimulated T4 secretion in both seasons and T3 secretion during the LD period.

The seasonal differences in LPS action are difficult to explain. However, it was stated that a seasonal switch in histone deacetylase (HDACs, mostly *Hdac4* and *6*) gene expression in the hypothalamus may participate in seasonal changes in *Dio2* and *Dio3* gene expression in rats [56]. It is also known that LPS can also influence several HDACs at the mRNA level (*Hdac1*, *4*, *5*, *7*) in murine bone marrow-derived macrophages [57]. Moreover, Wu et al. [58] stated that the mechanism of the HDAC2/c-Jun signaling network regulates the LPS-induced immune response in macrophages. It seems that a season-HDAC-LPS relationship is one of the key factors explaining the LPS photoperiod-dependent differences obtained in the present study.

The T3 and T4 concentrations in the blood after LPS treatment can be also dependent on their tissue uptake and their metabolism tissue metabolism. When male pigs were treated with LPS [59], *DIO2* and *DIO3* relative gene expressions were tissue-dependent, whereas in the liver, both *DIO2* and *DIO3* expressions were increased after LPS administration. In the spleen, *DIO2* expression was increased and *DIO3* expression was decreased. The increased *DIO2* gene expression was also observed in the lymph node and thymus. Such observations are consistent with the observations for the LD season from the present, which may suggest increased DIO2 activity that prevents a further decrease in T3 concentration.

## 4. Materials and Methods

### 4.1. Animals and Experimental Design

The procedures connected with animal welfare and caring were approved by the 3rd Local Ethical Commission of Warsaw University of Life Sciences—SGGW (Warsaw, Poland, authorization no. 56/2013). The ewes were in good condition and were kept under constant veterinary care.

The experiment was conducted on 48 adult (about 2-year old) female blackface ewes during two different photoperiods: the short-day (SD) period (day:night 8:16; autumn-winter time with short days and long nights; in Poland, the shortest day is around the 21st of December, so the experiment was conducted in December) and the long-day (LD) season (day:night 16:8; spring-summer time with long days and short nights; in Poland, the longest day is around the 21st of June, so the experiment was conducted in June). The animals were maintained indoors under natural lighting conditions (latitude 52° N, 21° E) in individual pens. The stress of social isolation was limited by visual contact with other members of the flock. The animals were acclimated to the experimental conditions for one month. The animals were fed a consistent diet of commercial concentrates with hay and water available ad libitum according to the recommendations of the National Research Institute of Animal Production [60].

In the SD season experiment, the stage of the oestrous cycle of ewes was synchronized by the Chronogest^®^ CR (Merck Animal Health, Boxmeer, The Netherlands) method using an intra-vaginal sponge impregnated with 20 mg of a synthetic progesterone-like hormone. All ewes had Chronogest^®^ CR sponges placed for 14 days. After sponge removal, the ewes received an intramuscular (*i.m.*) injection of 500 IU of pregnant mare’s serum gonadotropin (PMSG) (Merck Animal Health, Boxmeer, The Netherlands). The experimental procedure began 24 h following PMSG injection, so the ewes were in the follicular phase of the oestrous cycle. The same synchronization scheme was presented by Przybył, et al. [61]. During the LD period, animal synchronization was not required, as the animals were in seasonal anoestrous.

In both experiments, the animals were randomly divided into four groups, *n* = 6 in each: control (C), LPS injection to induce immune stress (LPS), leptin injection (LEP) and LPS and leptin injection (LPS + LEP). The LPS from *Escherichia coli* 055:B5 (Sigma-Aldrich, St. Louis, MO, USA) was dissolved in saline and injected into the jugular vein at a dose of 400 ng/kg of body mass [62]. The recombinant sheep leptin (Protein Laboratories Rehovot (PLR) Ltd., Rehovot, Israel) was also dissolved in saline and was injected 30 min after LPS treatment at a dose of 20 μg/kg of body mass (based on the dose used for growing beef heifers according to Maciel et al. [63]). The control animals received an equivalent volume of saline (0.9% *w*/*v* NaCl) (Baxter, Deerfield, IL, USA) at the moment of LPS and leptin injection (Table 1).

During the experimental day, the blood sampling was performed every 30 min, starting 1 h before and continuing 3 h after LPS/saline treatment (nine time points: −1 h, −0.5 h, 0 h, 0.5 h, 1 h, 1.5 h, 2 h, 2.5 h and 3 h). Blood samples were collected into tubes with ethylenediaminetetraacetic acid (EDTA) or heparin as a stabilizer and centrifuged, and the obtained blood plasma samples were immediately frozen and stored at −20 °C.

### 4.2. Blood Hormones Concentration Assay

#### 4.2.1. Leptin

The leptin plasma concentration was analyzed by a highly specific ovine leptin radioimmunoassay (RIA) using a high-affinity anti-ovine leptin rabbit antibody, anti-rabbit-γ-globulin antisera (double-antibody method) and a recombinant ovine leptin standard, as described by Delavaud et al. [64]. The sensitivity of the assay was 0.3 ng/mL, and the intra- and inter-assay coefficients of variation were 2.4 and 10.7%, respectively.

#### 4.2.2. Insulin

The plasma insulin concentration was determined using a commercial RIA kit (Porcine Insulin RIA kit; cat no PI-12K; Linco Research, St. Louis, MO, USA). The inter-assay coefficient of variation was 8.7%. The sensitivity of the assay was 1.611 μU/mL. The same kit for the sheep blood analysis was used by Catunda et al. [65].

#### 4.2.3. Thyroid Hormones

The thyroid hormone (T4 and T3) contents in blood were analyzed with the use of commercial RIA Kits—the Total T3 RIA Kit (cat. no IM1699) and the Total T4 RIA Kit (cat. no IM1447)—according to the manufacturer’s instructions (Beckman-Coulter; IMMUNOTECH, Prague, Czech Republic). The sensitivity of the kits was 0.26 and 10.63 nmol/L for T3 and T4, respectively. The intra- and inter-assay coefficients of variation were 6.3 and 7.7% for T3 and 3.29 and 7.53% for T4, respectively. The same kits were used by Bivolarski et al. [66].

#### 4.2.4. Cortisol

The cortisol concentration was determined by RIA according to Kokot and Stupnicki [67] using rabbit anti-cortisol antisera (R/75) and an HPLC-grade cortisol standard (Sigma-Aldrich, St. Louis, MO, USA). The assay sensitivity was 1 ng/mL, and the intra- and interassay coefficients of variation were 9 and 12%, respectively.

### 4.3. Statistical Analysis

Statistical analysis was performed using Statistica ver. 13.3 (TIBCO Software Inc., Palo Alto, CA, USA). Four types of statistical analysis were performed:

1. Repeated measured analysis of variance (ANOVA) followed by a post-hoc Fisher’s test, with the animal as a dependent factor for time point (nine time points: −1 h, −0.5 h, 0 h, 0.5 h, 1 h, 1.5 h, 2 h, 2.5 h and 3 h) comparisons for each experimental group separately (*n* = 6 for each time point, as there were six animals in each group);
2. Student’s *t*-test for the comparison between groups treated with the same factor (C, LPS, LEP and LPS + LEP) but in different seasons (SD vs. LD) for each time point separately (*n* = 6);
3. Student’s *t*-test for a general comparison between seasons (SD vs. LD) at a 0 h time point—after a one-hour adaptation but before any factor injection (*n* = 24, as there were 24 animals in each season);
4. Two-way ANOVA, with LPS and leptin injections as the main factors, followed by a post-hoc Fisher’s test for a comparison between the experimental groups (C vs. LPS vs. LEP vs. LPS + LEP) at selected time points (0 h, 0.5 h, 1 h, 2 h and 3 h) for each season separately (*n* = 6).

The results for all the above-mentioned analyses were considered statistically significant at *p* ≤ 0.05, and a tendency was observed when 0.1 > *p* > 0.05. The ANOVA analysis was performed after its two assumptions; the normality (Shapiro-Wilk’s test) and homogeneity of variances (Levene’s test) were checked. For the repeated measured ANOVA, sphericity assumption (Mauchly’s test) was also checked. The post-hoc test was performed only if the *p*-value from the ANOVA test was significant or if there was a tendency.

All data are presented as the mean ± standard error (SE).

## 5. Conclusions

In summary, in ewes, the photoperiod is one of the main factors influencing the basal blood concentrations of several hormones with higher values of leptin insulin and thyroid hormones during the long-day season. Additionally, acute inflammation’s effect on blood hormones, such as cortisol, insulin and thyroid hormones, is photoperiod-dependent in ewes. The endotoxemia may also exert an influence on leptin blood concentration, regardless of the examined photoperiod. The effects of leptin alone on hormone blood concentrations are rather limited; however, leptin can modulate the influence of LPS on insulin or thyroxine in a photoperiod-dependent way.

In conclusion, the obtained results showed that acute inflammation effects are strictly photoperiod-dependent in animals with marked seasonal differences, which may find an application in veterinary medicine. However, it cannot be said whether leptin can be used as a single-dose anti-inflammatory drug in acute inflammation, so further research in this field could be of interest not only to animal sciences but also to human medicine.

## Figures and Tables

**Table 1:** Experiment scheme.

No.	Group	Number of Animals	Experimental Factor	LPS Dose [ng/kg of Body Mass]	Leptin Dose [μg /kg of Body Mass]
**Experiment 1—Short-Day Season**					
1.	Control	6	NaCl	0	0
2.	LPS	6	LPS	400	0
3.	LEP	6	Leptin	0	20
4.	LPS + LEP	6	LPS + Leptin injected 30 min after LPS	400	20
**Experiment 2—Long-Day Season**					
5.	Control	6	NaCl	0	0
6.	LPS	6	LPS	400	0
7.	LEP	6	Leptin	0	20
8.	LPS + LEP	6	LPS + Leptin injected 30 min after LPS	400	20

LPS—lipopolysaccharide from Escherichia coli 055:B5 (Sigma-Aldrich, St. Louis, MO, USA); Leptin—the recombinant sheep leptin (Protein Laboratories Rehovot (PLR) Ltd., Rehovot, Israel).

## Data Availability

The data presented in this study are available upon request from the corresponding author.
